# Can healthcare professionals benefit from appreciative inquiry? Evidence from quasi-experiments to improve job resources, work engagement and vigour

**DOI:** 10.1108/JHOM-12-2025-0886

**Published:** 2026-08-04

**Authors:** David van de Ven, Martijn Hendriks

**Affiliations:** Erasmus Happiness Economics Research Organisation (EHERO), Erasmus University Rotterdam, Rotterdam, Netherlands; University of Johannesburg, Johannesburg, South Africa

**Keywords:** Appreciative inquiry, Work engagement, Healthcare, Job resources, Team crafting

## Abstract

**Purpose:**

To evaluate: (1) the effectiveness of team-based (quasi-)experiments developed with appreciative inquiry on job resources, team crafting, work engagement and vigour among healthcare professionals and (2) associations between the experimental process and changes in these outcomes.

**Design/methodology/approach:**

In a Dutch elderly care organisation, 174 participants and 495 non-participants were invited to complete baseline and three-month follow-up surveys. Participating teams designed a total of five quasi-experiments targeting job resources. Effectiveness was analysed using Kernel-based propensity score matching, and associations between the experimental process and outcome changes were analysed using linear regression adjusted for baseline values and covariates.

**Findings:**

Participation in experiments increased colleague social support. Workers more satisfied with experiment-related supervisor support showed greater improvements in work engagement, vigour, development opportunities, feedback about work and social support and coaching from supervisors. Workers more satisfied with the experiment’s effects and its alignment with their needs reported improved social support from supervisors.

**Practical implications:**

Using appreciative inquiry to collaboratively develop team-based experiments aimed at enhancing job resources may improve colleague social support among healthcare professionals. When supervisors provide more experiment-related support, work engagement and vigour might also improve.

**Originality/value:**

This is the first quasi-experimental study on the effectiveness of appreciative inquiry implemented within a regular work practice of healthcare workers. This design facilitated stronger causal inferences on the conditions under which appreciative inquiry might be effective, and allowed conclusions about the effects expected in similar work contexts.

## 1. Introduction

The sustainable employability of healthcare professionals is under pressure. The already high workload of these professionals increased since the COVID-19 pandemic because of changes in the working environment and staff shortages (Doleman *et al.*, 2023). An international scoping review suggested that pre-existing mental health problems of healthcare professionals increased in variation, severity and prevalence post-pandemic (Van den Broek *et al.*, 2023). As staff shortages persist, promoting work engagement – especially vigour – has become a priority for healthcare organisations. Engaged workers feel energetic, enthusiastic and immersed in their work (Schaufeli *et al.*, 2002). Vigour refers to high energy, feeling strong and fit, not easily fatigued, resilience and persistence. Strengthening work engagement and vigour counters work-related stress, improving performance and workability, as demonstrated in various occupational groups (Van Scheppingen *et al.*, 2015; Rongen *et al.*, 2014), including healthcare professionals working in intensive care settings (Van Mol *et al.*, 2018). This underlines the need for suitable interventions for healthcare workers to improve their work engagement and vigour, given the demanding conditions they are exposed to. Against this background, the Dutch government, through the Netherlands Organisation for Health Research and Development, initiated the programme “Innovative interventions to strengthen the vigour of employees”. Within this programme, an appreciative inquiry-based intervention involving team-based experiments was implemented in a Dutch elderly care organisation by an external intervention provider. As part of the programme, the outcomes of this intervention were independently evaluated by the authors of this study. The present study reports the findings from this intervention.

### 1.1 Background

Whereas traditional occupational interventions follow the “medical disease model” by targeting employees with (mental) health problems or those at risk, amplition focuses on promoting the well-being of all employees, including work engagement and vigour (Schaufeli and Salanova, 2010). However, systematic reviews and meta-analyses (Knight *et al.*, 2017, 2019) show limited effectiveness of interventions in promoting work engagement and vigour across various occupational contexts, including healthcare organisations. These studies underline the need to better understand the association between the implementation process and outcomes. Positive effects were more pronounced for group-level interventions and job crafting interventions, in which workers proactively optimise their job resources so they better align with their needs and preferences. The relevance of group-level approaches is especially evident in healthcare settings, where interprofessional collaboration is a core aspect of daily practice and essential for improving healthcare delivery outcomes (Wei *et al.*, 2022). However, it remains unclear whether team-based interventions that collaboratively strengthen job resources can improve work engagement and vigour under high job demands, and whether their effectiveness depends on the implementation process.

A promising approach for healthcare workers is appreciative inquiry, a strengths-based change method that emphasises what works well in organisations and teams and aims to build on these successes rather than focusing solely on solving problems (Cooperrider *et al.*, 2003; Armstrong *et al.*, 2020). This generates more positive energy and stimulates motivation and collaboration. The appreciative inquiry process typically follows the “4D-cycle”: (1) discover strengths, (2) dream about the desired future, (3) design of strategies and plans to realise the shared vision and ambitions, and (4) destiny, referring to implementation and adaptation in practice. The approach aligns with the collaborative nature of healthcare work and provides a structured bottom-up framework for co-developing and implementing interventions directly in daily practice. The systematic review by Merriel *et al.* (2022) on the impact of appreciative inquiry in healthcare and the integrative review by Cho and Ardichvili (2024) point out that, while the approach is widely applied and studied in other areas such as organisational development, it is increasingly studied within healthcare. This reflects the growing attention to appreciative inquiry for this group. Cho and Ardichvili (2024) provide tentative evidence for positive outcomes such as an increased sense of belonging, improved collaboration and the development of a shared vision, findings consistent with those reported by Merriel *et al.* (2022). These outcomes are mainly based on qualitative and observational studies, which limits the ability to make causal claims about effectiveness and to disentangle whether effectiveness depends on the implementation process. A quasi-experimental approach is better suited to make inferences about causality while remaining feasible in real-world workplace settings, offering insights that add to the current evidence base.

### 1.2 Theoretical considerations

The job demands-resources (JD-R) model (Schaufeli and Taris, 2014) served as the primary theoretical framework guiding the interventionists in understanding how team-based experiments developed through appreciative inquiry may improve vigour and work engagement by strengthening job resources and promoting team crafting. Team crafting is a bottom-up process inspired by the JD-R model in which team members jointly reshape their work by increasing job resources and challenging demands, while reducing hindering demands (Tims *et al*., 2013, 2022). Team crafting was used within the appreciative inquiry approach to align the experiments more closely with team-specific priorities, increasing the likelihood that participation enhances the targeted job resources. In addition, social identity theory (SIT) suggests that individuals derive part of their identity from the social group they belong to, which shapes their attitudes and behaviours (Tajfel and Turner, 1986; Haslam *et al.*, 2009). Participation in these collaborative experiments may therefore activate a shared team identity, which in turn strengthens social job resources and increases commitment to the experiment, thereby enhancing its overall effect on job resources. This is expected to activate the motivational pathway described in the JD-R model (Schaufeli and Taris, 2014), ultimately improving work engagement and vigour.

The extent to which the team-based experiments improve work engagement and vigour is expected to depend upon the processes through which these experiments unfold. In line with this, Nielsen and Randall (2013) and Nielsen *et al.* (2022) argue that organisational interventions should be understood as complex and emergent processes rather than static ones, with outcomes shaped by design, implementation, context and participants’ mental models. This implies that process characteristics may influence the mechanisms through which job resources are strengthened and the motivational pathway is triggered. Various process characteristics and participants’ perceptions of these may be relevant, ranging from supervision by interventionists to involvement by team members. The use of action plans requires particular attention because the literature offers two competing mechanisms. Action plans can facilitate effective interventions by specifying concrete activities and responsibilities linked to the intended outcomes (Nielsen, 2013). While adherence may support implementation as intended and strengthen targeted job resources, adaptation during implementation may also enhance effectiveness by allowing teams to respond to emerging needs. Despite the recognised importance of the intervention process, more sophisticated analyses of the interaction between the process and outcomes remain limited (Nielsen, 2013; Nielsen *et al.*, 2022).

### 1.3 Aims of the present study

Against this background, we aimed to evaluate, within a Dutch elderly care organisation: (1) the effectiveness of team-based (quasi-)experiments developed with appreciative inquiry on job resources, team crafting, work engagement and vigour of healthcare professionals, and (2) the extent to which the experimental process (satisfaction with process aspects and adherence to action plans) is associated with changes in these outcomes.

## 2. Methods

### 2.1 Study design and intervention procedure

After funding approval, the purpose and relevance of the intervention programme were discussed with managers and team supervisors. An elderly care organisation was deliberately selected as the study population because job profiles and work activities are relatively homogeneous across teams (mostly nurses, care assistants, client well-being coordinators, client support workers and supervisors), enhancing comparability in the evaluation of programme effects across teams. All teams within the organisation were eligible to participate, and no pre-selection was made by the researchers. The researchers and project coordinator of the elderly care organisation decided not to randomise participation in the programme and the team-based experiments, as this was not feasible in practice and did not align with the autonomy of workers inherent to appreciative inquiry. Nine self-selected teams with a total of 174 workers joined the programme. On-call workers, holiday workers and interns were excluded from the study. The programme consisted of the following five steps:

1. In March 2023, workers in the experimental group were invited by the researchers to complete a web-based survey with questions on work engagement, vigour, job resources and team crafting. The results were shared with teams as input for step two.
2. Guided by the interventionists, each team individually proceeded with the first three phases of the 4D-cycle: discovery (strengths and competences), dream (desires for job resources to enhance vigour and work engagement) and design (a (quasi-)experiment). A team-crafting approach was applied throughout, focusing on collaboratively increasing job resources. Five experiments were developed, with some teams using the same experiment. These five experiments included: (1) sharing daily what team members valued in their work to enhance social support; (2) the same as experiment 1, with an added morning check-in to clarify work-related expectations; (3) reflecting on challenging work situations to foster development opportunities; (4) clarifying expectations for regular work assignments and exchanging feedback to strengthen feedback about work and (5) sharing work-related knowledge to strengthen development opportunities. Each team created an action plan, supervised by interventionists and participated only in their own experiment (see Appendix Table A1).
3. Before the start of the experiments in September, 174 programme participants and 495 workers from teams that chose not to participate in the programme were invited to complete a survey on work engagement, vigour, job resources and team crafting. This survey is the baseline measurement in the current study.
4. The experiments were carried out for three months. A three-month period was the time available within the grant period for the implementation of the experiments. The selected experiments were expected to have immediate effects because they could be directly implemented within regular work practice and their low-barrier content (e.g. sharing daily what team members valued) required no start-up period to take effect. The effects were expected to persist, or even grow, over time because the experimental activities occurred repeatedly, ranging from daily to once per month. Moreover, this period directly follows the design phase when teams exhibit high shared commitment to enacting their co-created plans, which is expected to facilitate that the majority of the effects occur within this three-month period. Participation was voluntary, as this aligns with the emphasis on autonomy inherent to appreciative inquiry.
5. After three months, both groups were invited to complete the last questionnaire on the work-related measures.

### 2.2 Measurements

#### 2.2.1 Work engagement

Work engagement was measured at baseline and after three months with the three-item (α = 0.86) Utrecht Work Engagement Scale (UWES-3) (Schaufeli *et al.*, 2019). Workers were asked how often they: (1) feel bursting with energy at work, (2) are enthusiastic about their work and (3) are immersed in their work. Answer categories ranged from 1 (never) to 7 (always). Self-reported change in work engagement was also assessed after three months: experiment participants reported the experiment’s impact, while non-participants reported any change over the past three months. This was asked on a scale from −2 (very negative) to 2 (very positive), with 0 indicating no impact of the experiments or no change.

#### 2.2.2 Vigour

This was measured using the six items from the vigour dimension of the Utrecht Work Engagement Scale-17 (UWES-17) (Schaufeli *et al.*, 2002) (α = 0.90). The items assessed how often workers: (1) feel bursting with energy at work, (2) feel strong and vigorous at their job, (3) feel like going to work when they got up in the morning, (4) can continue working for very long periods of time, (5) are mentally resilient and (6) persevere at work, even under pressure (1 = never, 7 = always). After three months, self-reported changes in vigour due to the experiments (for participants) or over any change in vigour (for non-participants) were measured (same −2 to 2 scale).

#### 2.2.3 Job resources

Job resources were selected based on their established associations with work engagement (Schaufeli and Bakker, 2020) and consensus between researchers, workers and the project coordinator. These were measured at baseline and after three months using a 7-point frequency scale (1 = never, 7 = always). Social support from colleagues (α = 0.94) and supervisors (α = 0.96) was assessed by asking about: (1) reliance in difficult situations, (2) requesting help and (3) feeling valued. Supervisor coaching (α = 0.95) was measured based on: (1) satisfaction, (2) understanding, (3) appreciation, (4) problem-solving support and (5) approachability. Feedback about work (α = 0.93) included: (1) purpose, (2) performance and (3) outcomes. Development opportunities (α = 0.93) covered: (1) developing strengths, (2) grow within work and (3) learning new things. These items were derived from the Questionnaire on the Experience and Evaluation of Work (QEEW) developed by Van Veldhoven *et al.* (2002).

#### 2.2.4 Team crafting

Team crafting was measured using eight items from the Dutch job crafting scale (Tims *et al.*, 2012), adapted for the team level (Tims *et al.*, 2013). It covers four dimensions: structural and social job resources, challenging job demands and hindering job demands. Workers were asked how often team members (1) utilise capacities, (2) decide together, (3) rotate tasks, (4) share emotionally demanding tasks, (5) give feedback, (6) seek advice, (7) initiate new projects and (8) learn about developments (α = 0.93).

#### 2.2.5 The process of experimenting

This was assessed in the three-month follow-up survey with two sets of questions. First, workers reported on their participation: whether they joined the experiment, session frequency, average duration use of tools provided by the interventionists to support in achieving the intended outcomes (e.g. example questions for team dialogues), and reasons for non-participation or deviation from the action plan. Adherence was classified as complete, partial or none. Complete adherence meant following the planned session frequency and duration, and often or always using the tools provided by the interventionists. Partial adherence indicated that some, but not all, elements were followed. Non-adherence meant none of these aspects were followed; instead, they adapted their frequency, duration and tools during the experiment to better meet their possibilities and preferences. Complete and partial adherence were combined for analysis (as only four workers fully adhered), with non-adherence as the reference category. Second, workers rated their satisfaction (1 = very unsatisfied to 5 = very satisfied) with aspects of the experimental process: (1) supervision by the interventionists, (2) alignment with wishes and needs, (3) supervisor support, (4) experiment effect, (5) usefulness, (6) integration with work, (7) colleagues’ involvement and (8) organisational communication. These process aspects were inspired by factors identified as important in different phases of the intervention process (Nielsen *et al.*, 2022) and were selected through consensus among researchers, workers and the project coordinator to ensure relevance to the current context.

#### 2.2.6 Covariates

Sociodemographic and employee data were obtained from the organisation, including gender, birth year, contract type, job title, weekly working hours, workdays per week and years of service. Birth year was converted into age. Job titles were used to determine socioeconomic status via the International Socio-Economic Index (ISEI-08: range 16-90) (Ganzeboom *et al.*, 1992; Ganzeboom, 2010), with positions coded according to the ISCO-08 system.

### 2.3 Statistical analysis

#### 2.3.1 Descriptive analysis

Baseline characteristics were compared among (1) workers who participated in an experiment, (2) workers who participated in the programme but not in the experiments and (3) workers who did not join the programme. Logistic regression analysis was performed to assess determinants of dropout after three months.

#### 2.3.2 The effectiveness of quasi-experiments

Workers who participated in an experiment were compared with (1) programme participants who chose not to participate in the experiment and (2) workers outside the programme. Because participation was voluntary, selection bias was a potential threat to causal inference. To mitigate this, Kernel-based propensity score matching with automatic bandwidth selection was employed to reduce selection bias due to voluntary participation (Becker and Ichino, 2002). Propensity scores were based on all baseline characteristics, except gender when comparing to the non-experimenting programme group (no males in this group). Standardised mean differences (SMDs) were calculated both before and after matching to evaluate how much imbalance between groups remained (see Table A6, Appendix A). Residual imbalances (SMD >0.1) were further adjusted using additional covariates (Nguyen *et al.*, 2017). Despite these measures to analyse and reduce selection bias, residual imbalance may still remain, for example due to unobserved factors or subtle differences not captured in the baseline characteristics. Nevertheless, these measures increase causal claims that can be made about the effectiveness of team-based experiments, while maintaining ecological validity within a real-world care setting. Ultimately, a two-step analysis was conducted. First, crude linear regression models were applied, adjusting for baseline outcome variables to account for dependency between pre- and post-intervention observations and to mitigate regression to the mean (Vickers and Altman, 2001). Second, the Kernel-based propensity score models were applied. The average treatment effect (ATE) was used when comparing to non-experimenting programme participants, as the experiments were tailored to these workers (Benedetto *et al.*, 2018). The average treatment effect on the treated (ATT) was used when comparing to workers outside the programme, as it is more relevant for targeted interventions.

#### 2.3.3 Associations between the experimental process and changes in outcomes

Linear regression models assessed the extent to which (1) adherence to the action plan and (2) satisfaction with the eight aspects related to the experiments were associated with three-month changes in work engagement, vigour, job resources and team crafting. Each of these associations was adjusted for the corresponding baseline outcome variable and for covariates identified via backward elimination as the strongest predictors of change in the outcome.

#### 2.3.4 Robustness checks

Robustness checks included two components. First, alternative outcomes (self-reported changes in work engagement and vigour) were used to assess both the effectiveness of the experiments and the moderating impact of the experimental process. Second, different matching ratios (1:2 and 1:5 nearest-neighbour) were applied to test the robustness of the effectiveness estimates, with 1:2 considered as optimal for balance and higher ratios improving precision at the cost of bias (Austin, 2010).

### 2.4 Ethical considerations

This study was approved by the Internal Review Board of Erasmus School of Economics (ETH2223-0440). Prior to completing each questionnaire, participants were informed about the study and how their data would be handled. They were then asked to provide informed consent, and only those who gave their consent were included in the study. The data and code used in this study are publicly available in the DANS Data Station, under the following DOI: https://doi.org/10.17026/SS/EBAGLC.

## 3. Results

### 3.1 Study population

**Figure 1:**
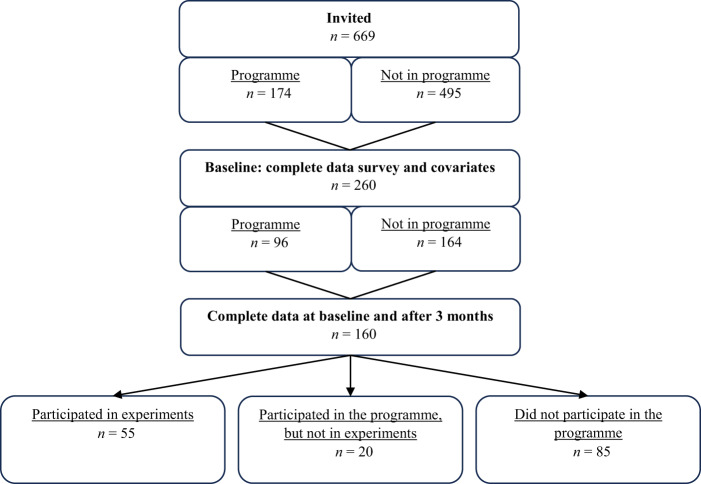
Flowchart of participants in the study

Baseline and covariate data were available for 260 workers, with 160 (62%) completing the three-month follow-up (Figure 1). Of these, 55 participated in both the programme and experiments, 20 in the programme only, and 85 did not participate. Most participants were female or had permanent contracts (Table 1). On average, they had intermediate socioeconomic status, worked part-time and had 6–12 years of work experience. Workers generally had high work engagement, vigour and favourable job resources. Workers outside the programme were less likely to have a permanent contract and had lower socioeconomic status, as well as less social support from colleagues and supervisors, less feedback about work, and fewer development opportunities. Loss to follow-up was lower among those with permanent contracts, older workers and those with higher socioeconomic status. A higher loss to follow-up was found among workers who received more coaching and feedback (see Appendix Table A2).

**Table 1:** Baseline characteristics of workers in and outside the programme

	Participated in the programme				Did not participate in the programme (*n* = 85)	
	Participated in experiments (*n* = 55)		Did not participate in experiments (*n* = 20)			
	Mean (SD)	%	Mean (SD)	%	Mean (SD)	%
*Demographics*						
Female (ref. male)		96.4		100		88.2
Permanent (ref. temporary) contract		96.4		95.0		*85.9*
Age	47.6 (12.2)		44.35 (11.9)		47.3 (12.4)	
Socioeconomic status	50.6 (19.8)		43.7 (16.8)		*44.8* *(17.8)*	
Hours working per week	26.6 (5.6)		25.3 (6.9)		25.7 (7.2)	
Days working per week	3.78 (3.78)		3.70 (0.80)		3.71 (1.00)	
Years working in the organisation	11.5 (12.9)		6.6 (6.3)		10.5 (10.2)	
*Work-related characteristics (1. never to 7. always)*						
Work engagement	4.98 (0.87)		4.87 (1.13)		4.80 (0.97)	
Vigour	4.89 (0.88)		4.73 (1.10)		4.71 (0.96)	
Social support from colleagues	5.90 (1.04)		5.80 (1.24)		*5.56 (1.11)*	
Social support from supervisors	5.27 (1.51)		4.75 (1.56)		*4.76 (1.59)*	
Coaching by supervisors	4.75 (1.37)		4.35 (1.39)		4.50 (1.43)	
Feedback about work	4.36 (1.17)		4.05 (1.26)		*3.98* *(1.32)*	
Development opportunities	4.75 (1.21)		4.75 (1.45)		*4.34* *(1.23)*	
Team crafting	4.72 (1.08)		4.65 (1.31)		4.54 (1.06)	
*Characteristics of the experimental process*						
Adherence to action plan (ref. non-adherence)		63.6		N/a		N/a
Satisfaction (1 = very unsatisfied to 5 = very satisfied) with the:						
Supervision by interventionists	3.47 (0.71)		N/a		N/a	
Alignment with wishes and needs	3.09 (0.95)		N/a		N/a	
Support from supervisors of the team	3.11 (0.94)		N/a		N/a	
Individual- and team-related effect	3.20 (0.89)		N/a		N/a	
Usefulness for one's own work	3.16 (0.83)		N/a		N/a	
Ease to apply the experiment in practice	3.11 (0.99)		N/a		N/a	
Involvement by team members	3.40 (0.85)		N/a		N/a	
Communication by the organisation	3.29 (0.74)		N/a		N/a	

**Note(s):** SD = standard deviation, N/a = not applicable

Italic highlights characteristics that are statistically significantly different compared to workers who participated in experiments at *p* < 0.1

### 3.2 Characteristics of the conducted experiments

The highest number of workers participated in the experiment where team members discussed what they valued (*n* = 19), and in the experiment where they additionally clarified expectations to each other by asking questions (*n* = 12; see Appendix Table A1). Both experiments targeted social support from colleagues. Most workers participated in these experiments on a weekly basis for 15 minutes or daily for less than 20 minutes, respectively. In general, most workers participated with lower frequency and for a shorter duration each time than planned. The tools provided by interventionists were used occasionally at most. Among all the participants in the experiments, 64% were partially or fully compliant with the action plan (Table 1). On average, participants were slightly satisfied with aspects related to the experimental process, with the highest satisfaction reported for the supervision by the interventionists.

### 3.3 The effectiveness of quasi-experiments

As shown in Table 2, participation in the experiments was not associated with observed changes in work engagement and vigour after three months, both when adjusted for baseline values and in the models with Kernel matching. After matching, participation in the experiments was associated with a statistically significant 0.46-point increase (90% CI: 0.16; 0.76) in social support from colleagues compared to programme participants who did not participate in the experiments, and a 0.29-point increase (90% CI: 0.03; 0.55) compared to those who did not participate in the programme. These improvements in social support from colleagues were consistent with results from the crude model, although the effect compared to workers outside the programme just missed the 90% CI threshold. Several other differences observed in the baseline-adjusted model became non-significant after matching, including improvements in team crafting, development opportunities and a larger decrease in social support from supervisors compared to one of the comparison groups. No statistically significant effects were observed for other outcomes.

**Table 2:** Observed three-month changes in work engagement, vigour, job resources and team crafting

	Participants in experiments vs. workers in the programme who did not experiment		Participants in experiments vs. workers not in the programme	
	Model 1^a^	Model 2^b^	Model 1^a^	Model 2^b^
Observed change in outcomes after 3 months	*b (90%CI)*	*ATE (90%CI)*	*b (90%CI)*	*ATT (90%CI)*
Work engagement	0.12 (−0.16; 0.40)	0.15 (−0.14; 0.44)	−0.10 (−0.28; 0.08)	−0.12 (−0.33; 0.09)
Vigour	0.16 (−0.08; 0.41)	0.15 (−0.10; 0.41)	−0.14 (−0.30; 0.01)	−0.17 (−0.35; 0.00)
Social support from colleagues	*0.35* *(0.02; 0.68)*	*0.46* *(0.16; 0.76)*	0.23 (−0.00; 0.46)	*0.29* *(0.03; 0.55)*
Social support from supervisors	0.26 (−0.13; 0.65)	0.03 (−0.30; 0.36)	*−0.31* *(−0.61;−0.02)*	−0.16 (−0.58; 0.27)
Coaching by supervisors	0.17 (−0.21; 0.56)	−0.17 (−0.66; 0.33)	−0.08 (−0.33; 0.18)	−0.05 (−0.34; 0.24)
Feedback about work	0.27 (−0.15; 0.68)	0.19 (−0.26; 0.64)	0.12 (−0.18; 0.41)	0.11 (−0.22; 0.45)
Development opportunities	*0.41* *(0.05; 0.77)*	0.20 (−0.19; 0.58)	0.12 (−0.13; 0.37)	0.27 (−0.02; 0.57)
Team crafting	0.31 (−0.02; 0.64)	0.16 (−0.15; 0.47)	*0.23* *(0.03; 0.44)*	0.15 (−0.07; 0.36)

**Note(s):** CI = confidence interval, ATE = average treatment effect, ATT = average treatment effect on the treated

Italic highlights that the estimate is statistically significant at *p* < 0.1

a Associations are adjusted for the corresponding baseline outcome variable

b Kernel-based propensity score matching with automatic bandwidth selection + adjustment for a maximum of four covariates with the greatest imbalance

### 3.4 Associations between the experimental process and changes in outcomes

Adherence to the action plans for the experiments was not associated with a three-month change in work engagement, vigour, job resources and team crafting compared to non-adherence (Table 3). Satisfaction with support from team supervisors during the experiment was associated with higher improvements in work engagement (b = 0.23, 90%CI:0.08; 0.38), vigour (b = 0.18, 90%CI:0.04; 0.32), social support from supervisors (b = 0.57, 90%CI:0.33; 0.82), coaching by supervisors (b = 0.32, 90%CI:0.09; 0.55), feedback about work (b = 0.45, 90%CI:0.19; 0.71) and development opportunities (b = 0.31, 90%CI:0.10; 0.52) (Table 4). In addition, satisfaction with the alignment of the experiments with workers’ wishes and needs (b = 0.25, 90%CI:0.00; 0.50) and the individual- and team-related effect (b = 0.28, 90%CI:0.01; 0.55) was associated with improvements in social support from supervisors. No other associations were found between satisfaction with aspects related to the process of experiments and changes in outcomes.

**Table 3:** Associations between adherence to the experiment action plan and observed three-month changes in outcomes

	Adherence (complete or partial) vs. non-adherence
Observed change in outcomes after 3 months	*b (90%CI)*
Work engagement	0.02 (−0.29; 0.32)
Vigour	0.03 (−0.24; 0.30)
Social support from colleagues	0.05 (−0.27; 0.38)
Social support from supervisors	−0.15 (−0.64; 0.34)
Coaching by supervisors	−0.30 (−0.73; 0.13)
Feedback about work	0.44 (−0.04; 0.92)
Development opportunities	0.13 (−0.28; 0.54)
Team crafting	−0.08 (−0.40; 0.24)

**Note(s):** CI = confidence interval

Italic highlights that the estimate is statistically significant at *p* < 0.1. The associations are adjusted for the corresponding baseline outcome variable, gender, age, baseline weekly working hours and number of working days per week

**Table 4:** Associations between satisfaction with aspects related to the experiments and observed three-month changes in outcomes

	Satisfaction with aspects related to the experiments (1. Very unsatisfied to 5. Very satisfied)							
	Supervision by interventionists	Alignment with wishes and needs	Support from supervisors of the team	Individual- and team-related effect	Usefulness for one's own work	Ease to apply the experiment in practice	Involvement by team members	Communication by the organisation
Observed change in outcomes after 3 months	*b (90%CI)*	*b (90%CI)*	*b (90%CI)*	*b (90%CI)*	*b (90%CI)*	*b (90%CI)*	*b (90%CI)*	*b (90%CI)*
Work engagement	0.00 (−0.20; 0.20)	0.13 (−0.03; 0.28)	*0.23* *(0.08; 0.38)*	0.11 (−0.06; 0.28)	0.13 (−0.04; 0.31)	0.02 (−0.13; 0.16)	0.03 (−0.14; 0.19)	0.08 (−0.11; 0.27)
Vigour	−0.01 (−0.19; 0.16)	0.10 (−0.05; 0.24)	*0.18* *(0.04; 0.32)*	0.11 (−0.04; 0.25)	0.10 (−0.06; 0.25)	0.03 (−0.10; 0.16)	0.10 (−0.05; 0.24)	−0.03 (−0.20; 0.14)
Social support from colleagues	0.11 (−0.10; 0.32)	−0.05 (−0.22; 0.13)	0.07 (−0.10; 0.24)	0.02 (−0.16; 0.21)	0.12 (−0.07; 0.31)	0.14 (−0.03; 0.30)	0.02 (−0.16; 0.21)	−0.09 (−0.30; 0.12)
Social support from supervisors	0.00 (−0.32; 0.32)	*0.25* *(0.00; 0.50)*	*0.57* *(0.33; 0.82)*	*0.28* *(0.01; 0.55)*	0.13 (−0.15; 0.41)	0.14 (−0.10; 0.38)	0.00 (−0.27; 0.28)	0.28 (−0.03; 0.58)
Coaching by supervisors	−0.04 (−0.31; 0.24)	0.06 (−0.16; 0.28)	*0.32* *(0.09; 0.55)*	0.02 (−0.22; 0.26)	−0.11 (−0.35; 0.14)	−0.04 (−0.25; 0.17)	−0.13 (−0.36; 0.11)	0.13 (−0.14; 0.40)
Feedback about work	0.18 (−0.14; 0.49)	0.07 (−0.20; 0.34)	*0.45* *(0.19; 0.71)*	0.14 (−0.15; 0.43)	0.09 (−0.20; 0.39)	0.08 (−0.17; 0.32)	0.06 (−0.23; 0.34)	−0.09 (−0.41; 0.23)
Development opportunities	0.00 (−0.26; 0.27)	0.16 (−0.06; 0.37)	*0.31* *(0.10; 0.52)*	0.15 (−0.09; 0.39)	0.11 (−0.13; 0.35)	−0.01 (−0.22; 0.19)	0.20 (−0.03; 0.42)	0.15 (−0.11; 0.41)
Team crafting	0.02 (−0.19; 0.23)	0.00 (−0.17; 0.17)	0.11 (−0.05; 0.28)	0.03 (−0.16; 0.22)	0.09 (−0.10; 0.27)	0.08 (−0.09; 0.25)	0.17 (−0.02; 0.36)	−0.01 (−0.23; 0.21)

**Note(s):** CI = confidence interval

Italic highlights that the estimate is statistically significant at *p* < 0.1. The associations are adjusted for the corresponding baseline outcome variable, gender, age, baseline weekly working hours and number of working days per week

### 3.5 Robustness checks

The results mostly remained robust when Kernel matching was replaced by 1:2 and 1:5 nearest-neighbour matching (see Appendix Table A3). A positive effect of experiment participation on social support from colleagues was consistently observed, except when using 1:2 matching in comparison with non-programme participants, where the estimate fell below the 90% confidence level. The positive effect on development opportunities, compared to workers who did not participate in the programme, became statistically significant after matching with 5 neighbours. Other associations remained non-significant across the different matching techniques.

Participation in experiments was not associated with self-reported change in work engagement, consistent with observed changes (Appendix Table A3). Self-reported improvements in vigour were found in three out of six comparisons, particularly when using 1:2 matching, and partly contradicted the main analysis. Adherence was not associated with self-reported work engagement and vigour, which is also in line with the main analysis (Appendix Table A4). In addition, while only satisfaction with supervisory support during experiments was associated with observed improvements in work engagement and vigour, satisfaction with most aspects of the experimental process was associated with self-reported improvements in these outcomes.

### 3.6 Characteristics of the propensity score and matching procedures

The baseline characteristics explained between 13 and 19% of the variation in participation (see Appendix Table A5). The classification accuracy for the experimental group, compared to non-experimenting programme participants, was 79% (AUC = 0.79). For comparison with non-programme participants, the accuracy was 72% (AUC = 0.72). Both values are considered acceptable (Hosmer *et al.*, 2013). Post-matching statistics revealed that Kernel matching provided the best matches (see Appendix Table A6). Compared to 1:2 and 1:5 matching, Kernel matching resulted in the largest number of characteristics with SMDs that declined, came closest to 0, and fell below the accepted threshold of 0.1 (Stuart *et al.*, 2013).

## 4. Discussion

This study extends the evidence base on interventions aimed at improving work engagement and vigour among healthcare professionals by using a quasi-experimental design in a real-life work setting to evaluate the effectiveness of team-based experiments designed through appreciative inquiry and the role of the experimental process in influencing the outcomes. The findings show that colleague social support was the only job resource that improved after three months, which did not translate into improvements in work engagement and vigour. However, when workers were more satisfied with supervisor support during the experiment, additional job resources as well as work engagement and vigour showed greater improvements.

### 4.1 The effectiveness of the team-based experiments

Previous qualitative and observational studies (Cho and Ardichvili, 2024; Merriel *et al.*, 2022) suggest that appreciative inquiry may improve job resources and more distal outcomes such as work engagement. The present study extends this evidence by showing that collaborative team experiments developed through appreciative inquiry might improve colleague social support in the high-demand context of healthcare workers. Its quasi-experimental design in a real work setting allows for stronger causal inferences while maintaining ecological validity. The improvement in colleague social support can be explained by two main reasons. First, it aligns with the assumption derived from SIT (Tajfel and Turner, 1986; Haslam *et al.*, 2009) that participation in these collaborative experiments activates a shared team identity, which was expected to improve social job resources in particular. Second, the experiments with the most participants directly targeted social support by encouraging colleagues to share what they valued in each other’s work, which aligned with their team priorities. The team-based experiments targeting other job resources may also have been aligned with team priorities and activated shared commitment, but the smaller number of participants in those experiments likely limited their ability to produce measurable improvements in those targeted job resources. Future research should examine whether other job resources would also improve when these are more explicitly targeted through appreciative inquiry. A valid concern related to the quasi-experimental study design is that the observed improvement in colleague social support could result from participants’ awareness of being observed, expectancy effects, demand characteristics or simply from receiving attention. However, in this study, these threats are likely limited because no overall improvements were observed in any of the other targeted outcomes (vigour and work engagement) and job resources, making it less likely that the overall improvements in colleague social support can be attributed to such validity threats.

While it was expected from the JD-R model (Schaufeli and Taris, 2014) that improvements in job resources would trigger the motivational pathway to increased work engagement and vigour, our findings indicate that improvement in colleague social support alone was insufficient to result in improvements in work engagement and vigour. This is in line with previous research in other settings showing that job resource interventions do not consistently improve work engagement (Cifre *et al.*, 2011; Coffeng *et al.*, 2014). A possible explanation for this in this study is that the benefits of colleague social support for work engagement and vigour were outweighed by the high demands of healthcare workers. This is supported by other findings within this research as well as by other studies. Open-ended responses in this study revealed that healthcare workers reported time constraints and staffing issues as reasons for deviation from their action plan and non-participation, reflecting the high demands. Similarly, Shiri *et al.* (2023) found that inadequate staffing, high workload and time constraints limited the success of well-being interventions among health and social service workers. The discrepancy between self-reported improvements in vigour and the absence of observed changes in vigour may reflect participants’ tendency to overestimate the benefits of the experiments when asked about them directly, without fully considering the demands of their work context.

### 4.2 The role of the experimental process in the effectiveness

Building on prior research highlighting the need to evaluate the intervention process and its impact on well-being outcomes, including in healthcare (Nielsen, 2013; Nielsen *et al.*, 2022), our findings suggest that the effectiveness of team-based experiments developed through appreciative inquiry depends on the implementation process. In particular, when participants were more satisfied with the experiment-related support they received from their team supervisor, stronger improvements were observed in various job resources, work engagement and vigour. These findings reinforce the critical role of line managers in the intervention implementation process, consistent with Nielsen *et al.* (2010), Nielsen (2017), and Nielsen (2013). Through transformational leadership and shared values, line managers can enhance participation and outcomes. Similarly, Shiri *et al.* (2023) found that unsupportive leaders hindered the success of well-being interventions. This supports the assumption that higher satisfaction with the support from the team leader improves the commitment and the alignment between the experiments and the work context, enhancing the improvement in job resources. The findings further suggest that, even in high-demand work settings like healthcare, appreciative inquiry can contribute to work engagement and vigour when team leaders provide sufficient support during team-based experiments. This also indicates a limitation of the JD-R model, as it does not account for the boundary conditions under which job resources are improved.

Moreover, while this study and previous studies highlight the importance of supervisors in the implementation process (Nielsen *et al.*, 2010; Nielsen, 2017; Nielsen, 2013), improving the implementation process may also increase perceptions of overall supervisor support. Specifically, workers who were more satisfied with the effects of the experiments and how the experiment aligned with their needs and wishes reported greater improvement in overall social support from supervisors. This suggests that when workers are enabled by supervisors to create initiatives that benefit the team, they feel more supported.

Building on the highlighted importance of action plans for effective interventions (Nielsen, 2013), this study also aimed to examine whether the effects of the experiments were dependent on the extent to which participants adhered to the collaborative action plan. However, the effect of complete adherence could not be tested separately because only four respondents completely adhered to the plan. Therefore, complete and partial adherence were combined for analysis, which showed no differential effect compared to non-adherence. This finding and the low rate of complete adherence point to the need for ongoing adjustments based on the context, consistent with the iterative nature of action research (Kemmis *et al.*, 2014).

### 4.3 Strengths and limitations

This study has several strengths. The main strength is the quasi-experimental evaluation of team-based experiments implemented within the regular healthcare practice. This design allowed for stronger causal inferences than previous qualitative and observational studies on appreciative inquiry while maintaining ecological validity, thereby improving understanding of the conditions under which appreciative inquiry might be effective for healthcare workers and increasing the transferability of the results to high-demand team settings. A related strength is that the quasi-experimental study design enabled the use of multiple comparison groups, alternative measures of change in work engagement and vigour, and propensity score models, improving reliability and internal validity. Several limitations should also be noted. First, the modest sample size likely meant that only large effects reached statistical significance. Rather than indicating the absence of effects, non-significant results may therefore reflect more modest effect sizes, particularly for distal outcomes such as work engagement and vigour. The modest sample also limits generalisability. Second, the usual limitations of (quasi-)experiments conducted in field settings over multiple months apply. Notably, despite the use of carefully selected control groups, the treatment did not occur in isolation, raising the possibility of simultaneous changes in confounding factors that are unequally distributed between the intervention group and the control groups. In addition, although propensity score matching reduced imbalance between participants and non-participants and strengthened causal inferences compared to previous qualitative and observational studies on appreciative inquiry within healthcare, selection bias remained a threat to causal inference. The limited number of baseline covariates used may have resulted in residual imbalances between participants and non-participants, both measured and unmeasured. Yet, classification into the experimental and comparison groups based on the measured covariates was done correctly for most workers. Third, although appreciative inquiry allowed for direct implementation in regular work practice and the three-month participation period likely captured the majority of immediate changes in job resources, it does not allow conclusions about the sustainability of these effects. Future research is needed to examine the conditions under which these initial changes are maintained, strengthened or diminished.

## 5. Conclusion

The present study suggests that team-based experiments developed with appreciative inquiry can improve colleagues' social support for healthcare workers in collaborative teams exposed to high demands. These improvements do not automatically translate into higher work engagement or vigour if the implementation process is not adequately supported. However, when team supervisors provide strong support during the implementation process, the enhancement of job resources can lead to increases in both work engagement and vigour.

## 6. Recommendations

HR departments and policymakers in healthcare organisations can enhance job resources by implementing appreciative inquiry as an action-based, team-oriented approach in which members collaboratively develop, enact and refine initiatives to improve their work. Implementation should be monitored, with particular attention to supervisor support, which can be fostered through transformational leadership and shared values. Supervisors should actively support team members during the experiments by responding to their needs and facilitating alignment of initiatives so that both individuals and the team as a whole benefit. Researchers are encouraged to examine process factors as moderators of effectiveness, include relevant baseline variables and maximise response rates to strengthen causal inference using propensity score matching. Future research should adopt similar quasi-experimental designs to further examine the conditions under which appreciative inquiry and other initiatives aimed at improving job resources translate into work engagement and vigour, such as the work context and the implementation context. Future research should also explore whether longer periods of implementing and refining team initiatives further enhance job resources, and ultimately vigour and work engagement.

## Supplementary Material

Data supplement 1

## Notes

The supplementary material for this article can be found online.
