# Supplementary material for: Can healthcare professionals benefit from appreciative inquiry? Evidence from quasi-experiments to improve job resources, work engagement and vigour
Source: J Health Organ Manag. 2026 Aug 4;40(9):482–98. doi: 10.1108/JHOM-12-2025-0886 (PMC13428514; doi:10.1108/JHOM-12-2025-0886)
Supplement: Data supplement 1 [file jhom-12-2025-0886_suppl1.docx]

**APPENDIX**

**Table A1**. Characteristics of the plan and execution of experiments among programme participants with complete three-month data

| **Description experiment** | **Plan** | | | **Execution** | | | | |
| --- | --- | --- | --- | --- | --- | --- | --- | --- |
|  | **Job resource targeted** | **Frequency** | **Duration each time** | **n (% of invited)** | **Frequency** | **Duration each time** | **Frequency of example questions use** | **Reason for non-participation** |
| 1) On fixed times during the day (e.g. care handover or day’s end) team members meet up and ask each other questions on what they valued (e.g. 'What were you proud of?' or 'What gave you energy?’). Interventionists provided example questions. | Social support from colleagues | Daily | 15 minutes | 19 (61.3%) | Weekly | 15 minutes | Never/  sporadically | Lack of time/staffing issues |
| 2) Same as experiment 1 + a moment at the start of the day where team members clarify expectations to each other by asking questions (e.g. 'What do you hope to learn?' or 'What can I do for you?'). | Social support from colleagues | Daily | 20 minutes | 12 (91.3%) | Daily | <20 minutes | Occasionally | Not specified |
| 3) Team-members collect difficult situations they typically encounter in their work, and they organize sessions to discuss how to cope with each situation. | Development opportunities | 3 times in total | Not specified | 5 (45.5%) | Once | 1-10 minutes | N/a | Lack of time/not convinced of benefits |
| 4) Team members clarify expectations when they receive assignments from external clients, and they ask each other feedback by asking questions (e.g. 'What did you value?' or 'What do you need?'). | Feedback about work | For each assignment | Not specified | 10 (83.3%) | Occasionally | 1-10 minutes | Never/  sporadically | Not specified |
| 5) Team members organize knowledge sharing sessions where presentations are given, and relevant topics are discussed. | Development opportunities | 1 session/  month | 2 hours | 9 (100%) | Twice | 2 hours | N/a | N/a |

N/a=not applicable
The information on the executed frequency, duration, use of example questions, and reasons for non-participation is based on the most frequently chosen answer.

**Table A2**. Baseline characteristics associated with loss to follow-up after three months

|  | **Loss to follow-up** |
| --- | --- |
|  |  |
|  | OR (90% CI) |
| **Demographics** |  |
| Female (ref. male) | 2.16 (0.73;6.41) |
| Permanent (ref. temporary) contract | **0.37** (0.20;0.67) |
| Age | **0.98** (0.96;1.00) |
| Socioeconomic status | **0.99** (0.97;1.00) |
| Hours working per week | 0.99 (0.96;1.02) |
| Days working per week | 0.96 (0.76;1.21) |
| Years working in the organisation | 0.99 (0.97;1.01) |
|  |  |
| **Work-related characteristics** |  |
| Work engagement | 1.06 (0.85;1.32) |
| Vigour | 1.18 (0.94;1.49) |
| Social support from colleagues | 0.95 (0.79;1.15) |
| Social support from supervisors | 1.15 (0.99;1.33) |
| Coaching by supervisors | **1.20** (1.02;1.40) |
| Feedback about work | **1.23** (1.03;1.47) |
| Development opportunities | 1.12 (0.95;1.33) |
| Team crafting | 0.84 (0.69;1.03) |

CI=confidence interval, OR=odds ratio

Bold indicates that the logistic regression estimate is statistically significant at p<.1.

**Table A3.** Associations between participation in experiments and changes in outcomes after Kernel, 1:2, and 1:5 matching

|  | **Participants in experiments vs. workers in the programme who did not experiment** | | | **Participants in experiments vs. workers not in the programme** | | |
| --- | --- | --- | --- | --- | --- | --- |
|  | Kernel matching | 1:2 matching | 1:5 matching | Kernel matching | 1:2 matching | 1:5 matching |
| **Observed change in outcomes after 3 months** | ATE (90% CI) | ATE (90% CI) | ATE (90% CI) | ATT (90% CI) | ATT (90% CI) | ATT (90% CI) |
| Work engagement | 0.15 (-0.14;0.44) | -0.04 (-0.34;0.26) | 0.08 (-0.14;0.30) | -0.12 (-0.33;0.09) | -0.15 (-0.36;0.07) | -0.13 (-0.34;0.08) |
| Vigour | 0.15 (-0.10;0.41) | 0.05 (-0.18;0.27) | 0.13 (-0.10;0.36) | -0.17 (-0.35;0.00) | -0.17 (-0.35;0.00) | -0.12 (-0.29;0.06) |
| Social support from colleagues | **0.46** (0.16;0.76) | **0.50** (0.22;0.78) | **0.48** (0.18;0.78) | **0.29** (0.03;0.55) | 0.26 (-0.03;0.55) | **0.34** (0.07;0.61) |
| Social support from supervisors | 0.03 (-0.30;0.36) | 0.05 (-0.23;0.33) | 0.07 (-0.27;0.41) | -0.16 (-0.58;0.27) | -0.28 (-0.64;0.09) | -0.19 (-0.50;0.13) |
| Coaching by supervisors | -0.17 (-0.66;0.33) | -0.05 (-0.39;0.28) | -0.05 (-0.56;0.46) | -0.05 (-0.34;0.24) | -0.10 (-0.41;0.21) | -0.06 (-0.35;0.23) |
| Feedback about work | 0.19 (-0.26;0.64) | 0.30 (-0.01;0.60) | 0.28 (-0.17;0.73) | 0.11 (-0.22;0.45) | 0.15 (-0.21;0.52) | 0.16 (-0.14;0.46) |
| Development opportunities | 0.20 (-0.19;0.58) | 0.28 (-0.03;0.60) | 0.30 (-0.08;0.69) | 0.27 (-0.02;0.57) | 0.36 (-0.02;0.73) | **0.34** (0.06;0.63) |
| Team crafting | 0.16 (-0.15;0.47) | 0.23 (-0.06;0.52) | 0.24 (-0.07;0.56) | 0.15 (-0.07;0.36) | 0.12 (-0.10;0.34) | 0.17 (-0.01;0.35) |
|  |  |  |  |  |  |  |
| **Self-rated change due to experiments (for participants in experiments) or since September (for control group)** | ATE (90% CI) | ATE (90% CI) | ATE (90% CI) | ATT (90% CI) | ATT (90% CI) | ATT (90% CI) |
| Work engagement | 0.12 (-0.12;0.36) | 0.03 (-0.27;0.33) | -0.06 (-0.35;0.23) | 0.16 (-0.08;0.39) | 0.23 (-0.03;0.48) | 0.19 (-0.07;0.44) |
| Vigour | **0.34** (0.12;0.57) | **0.25** (0.02;0.48) | 0.22 (-0.00;0.43) | 0.19 (-0.09;0.47) | **0.31** (0.02;0.59) | 0.21 (-0.09;0.51) |

CI=confidence interval, ATE=average treatment effect, ATT=average treatment effect on the treated

Bold highlights that the estimate is statistically significant at p<.1. Covariates with imbalance (a maximum of 4) after matching were additionally added as control variables.

**Table A4.** Associations of action plan adherence, satisfaction with experimental aspects, and self-reported changes in outcomes

|  | **Self-reported change in work engagement** | **Self-reported change in vigour** |
| --- | --- | --- |
| **Adherence to the action plan for experiments** | b (90% CI) | b (90% CI) |
| Adherence (≥partially) (ref. non-adherence) | 0.20 (-0.12;0.52) | 0.28 (-0.02;0.58) |
|  |  |  |
| **Satisfaction with aspects related to the experiments (1. very unsatisfied to 5. very satisfied)** |  |  |
| Supervision by interventionists | **0.31** (0.11;0.50) | **0.31** (0.12;0.49) |
| Alignment with wishes and needs | **0.23** (0.07;0.39) | **0.28** (0.13;0.43) |
| Support from supervisors of the team | **0.29** (0.14;0.44) | **0.30** (0.15;0.45) |
| Individual- and team-related effect | **0.28** (0.12;0.45) | **0.31** (0.16;0.46) |
| Usefulness for one's own work | **0.24** (0.06;0.41) | **0.29** (0.13;0.45) |
| Ease to apply the experiment in practice | **0.25** (0.11;0.40) | **0.26** (0.12;0.39) |
| Involvement by team members | 0.15 (-0.02;0.32) | **0.19** (0.04;0.35) |
| Communication by the organization | 0.13 (-0.07;0.33) | 0.09 (-0.10;0.28) |

CI=confidence interval

Bold highlights that estimate is significant at p<.1. The associations are adjusted for the corresponding baseline outcome variable, gender, age, baseline weekly working hours, and number of working days per week.

**Table A5.** Associations between baseline characteristics and the odds to participate in experiments derived from multivariate models

| **Characteristics** | **Participants in experiments vs. workers in the programme who did not experiment** | **Participants in experiments vs. workers not in the programme** |
| --- | --- | --- |
|  | OR (90% CI) | OR (90% CI) |
| **Demographics** |  |  |
| Female (ref. male) | N/a | 4.09 (0.91;18.35) |
| Permanent (ref. temporary) contract | 0.90 (0.06;12.64) | **4.41** (1.02;19.02) |
| Age | 1.05 (0.99;1.12) | 1.00 (0.97;1.04) |
| Socioeconomic status | **1.05** (1.01;1.08) | **1.02** (1.00;1.04) |
| Hours working per week | 1.10 (0.97;1.25) | 1.04 (0.95;1.13) |
| Days working per week | 0.86 (0.32;2.32) | 1.00 (0.55;1.81) |
| Years working in the organisation | **1.07** (1.01;1.14) | 1.01 (0.98;1.04) |
|  |  |  |
| **Work-related characteristics** |  |  |
| Work engagement | 1.56 (0.36;6.70) | 0.88 (0.37;2.09) |
| Vigour | 0.63 (0.15;2.62) | 0.99 (0.41;2.35) |
| Social support from colleagues | 0.97 (0.49;1.92) | 1.23 (0.83;1.83) |
| Social support from supervisors | 1.41 (0.53;3.72) | **2.14** (1.20;3.82) |
| Coaching from supervisors | 1.33 (0.41;4.28) | **0.36** (0.17;0.74) |
| Feedback about work | 1.51 (0.74;3.07) | 1.36 (0.86;2.14) |
| Development opportunities | 0.57 (0.30;1.10) | 1.50 (0.98;2.30) |
| Team crafting | 0.72 (0.36;1.44) | 0.80 (0.51;1.24) |
| McFadden R^2^ (%) | 18.9% | 13.0% |
| Area Under the Curve | 0.79 | 0.72 |

CI=confidence interval, OR=odds ratio

Bold highlights that estimate is significant at p<.1.

**Table A6.** Standardized mean differences pre- and post-matching (Kernel, 1:2, 1:5) between experiment and comparison groups

|  | **Participants in experiments vs. workers in the programme who did not experiment** | | | | **Participants in experiments vs. workers not in the programme** | | | |
| --- | --- | --- | --- | --- | --- | --- | --- | --- |
|  | Standardized mean differences | | | | Standardized mean differences | | | |
|  | Raw | Kernel | 1:2 | 1:5 | Raw | Kernel | 1:2 | 1:5 |
| **Demographics** |  |  |  |  |  |  |  |  |
| Female (ref. male) | N/a | N/a | N/a | N/a | 0.31 | **-0.04** | **-0.07** | **-0.03** |
| Permanent (ref. temporary) contract | 0.07 | **-0.02** | 0.20 | **-0.08** | 0.37 | **0.04** | **-0.03** | **-0.03** |
| Age | 0.27 | **-0.07** | -0.16 | -0.15 | 0.03 | **-0.02** | **0.02** | **0.01** |
| Socioeconomic status | 0.38 | 0.21 | **-0.03** | **-0.03** | 0.31 | **-0.02** | **0.00** | **0.00** |
| Hours working per week | 0.21 | **0.05** | **0.05** | **-0.02** | 0.14 | **0.07** | **0.00** | **-0.03** |
| Days working per week | 0.11 | **0.07** | **-0.03** | **-0.08** | 0.09 | **-0.07** | -0.16 | **-0.01** |
| Years working in the organisation | 0.49 | **0.04** | -0.28 | -0.15 | 0.09 | **0.07** | **-0.03** | **-0.09** |
|  |  |  |  |  |  |  |  |  |
| **Work-related characteristics** |  |  |  |  | ` |  |  |  |
| Work engagement | 0.11 | 0.14 | 0.11 | 0.19 | 0.19 | **0.00** | **0.05** | **0.06** |
| Vigour | 0.16 | **0.09** | **0.01** | 0.16 | 0.20 | **0.01** | **0.07** | **0.03** |
| Social support from colleagues | 0.09 | -0.18 | -0.45 | **0.09** | 0.32 | **0.06** | **0.10** | 0.13 |
| Social support from supervisors | 0.34 | **0.01** | -0.42 | 0.13 | 0.33 | **-0.01** | **-0.03** | **-0.02** |
| Coaching by supervisors | 0.29 | **0.02** | -0.44 | **0.07** | 0.17 | **0.02** | **-0.04** | **-0.02** |
| Feedback about work | 0.26 | **0.01** | -0.45 | **0.09** | 0.31 | **0.07** | **0.06** | 0.11 |
| Development opportunities | 0.00 | -0.10 | -0.32 | **-0.04** | 0.33 | **0.04** | **0.01** | 0.11 |
| Team crafting | 0.06 | **0.05** | -0.27 | 0.23 | 0.17 | **0.07** | **0.03** | 0.12 |

N/a=not applicable
Bold highlights that the standardized mean difference is between -0.1 and 0.1 after matching.
